# Effect of sleep duration on child development in Fortaleza, Northeastern Brazil

**DOI:** 10.1016/j.jped.2024.09.002

**Published:** 2024-10-22

**Authors:** Shamyr Sulyvan Castro, Marcia Maria Tavares Machado, Luciano Lima Correia, Simone Farias-Antúnez, Pedro Lucas Grangeiro de Sá Barreto Lima, Sophia Costa Vasconcelos, Elisa Rachel Pisani Altafim, Marcia C. Castro

**Affiliations:** a Universidade Federal do Ceará (UFC), Departamento de Fisioterapia, Fortaleza, CE, Brazil; b Universidade Federal do Ceará (UFC), Departamento de Saúde Pública, Fortaleza, CE, Brazil; c Universidade Federal de Santa Catarina (UFSC), Departamento de Ciências da Saúde, Araranguá, SC, Brazil; d Universidade Federal do Ceará (UFC), Fortaleza, CE, Brazil; e Universidade de São Paulo (USP), Ribeirão Preto, SP, Brazil; f Harvard T. H. Chan School of Public Health, Boston, MA, USA

**Keywords:** Child development, Sleep duration, Cohort study

## Abstract

**Objective:**

The present study's objective is to assess whether sleep duration affects Early Childhood Development (ECD). A prospective cohort study was carried out with 278 mother-child dyads in the city of Fortaleza, northeastern Brazil, with data collection every 6 months.

**Method:**

The data used in this study are from the third (18 months) and fourth (24 months) survey waves. Information on sleep duration was collected using the Brief Infant Sleep Questionnaire (BISQ) and information on ECD using the Caregiver Reported Early Development Instrument (CREDI). Crude and adjusted regression models were run for each CREDI domain as an outcome with 5 % significance.

**Results:**

The authors found that after adjusting for maternal age and schooling, family income, and the presence of other children in the house, night sleep duration was associated with better ECD scores (cognitive: coef. 0.14; 95 % CI 0.04,0.24; language: coef. 0.10; 95 % CI 0.01,0.19; motor: coef. 0.10; 95 % CI 0.03,0.18; socio-emotional: coef. 0.16; 95 % CI 0.06,0.25; overall: coef. 0.14; 95 % CI 0.04,0.24), and the time awake at night associated with worse scores (cognitive: coef. -0.12; 95 % CI -0.23,0.02; motor: coef. -0.09; 95 % CI -0.17,-0.01; socio-emotional: coef. -0.11; 95 % CI -0.21,-0.01; overall: coef. -0.11; 95 % CI -0.21,-0.01).

**Conclusions:**

Nocturnal sleep duration affects ECD as expressed in all CREDI scores (cognitive, language, motor, social-emotional, and overall).

## Introduction

The COVID-19 pandemic affected health in varied ways, such as mental health,^1^ levels of physical activity;^2^ and changes in sleep quality for children.^3^ A review found that children's sleep was affected in various ways during the COVID-19 isolation period, leading to changes in sleep routines, patterns, and disturbances. The results indicated increased sleep duration, but findings on sleep latency were inconclusive.^4^ Sleep duration is a known health-related factor and sleeping for less than the recommended hours can lead to general health problems, worse quality of life, and cardiovascular, metabolic, pulmonary, allergy/atopy, and obesity-related conditions among children and adolescents in the long term.^5^ Although little discussed, short sleep duration is a relatively frequent problem, occurring in 33.3 % of children between 1 and 2 years of age, with variations according to ethnicity, income, and parents' educational level.^6^

Between 1 and 2 years of age is the period in which Early Childhood Development (ECD) takes place, which is understood as the children's cognitive, physical, language, motor, social, and emotional development, between conception and age 8.^7^ It is important to highlight that this is a period in people's lives in which a decrease in sleep can lead to impaired memory development, lack of emotional regulation, and inadequate levels of cognition^8^ and motor skills.^9^

Studies conducted during non-pandemic scenarios have shown that higher child developmental scores are associated with sex, caregiver education, socioeconomic status, and stimulation.^10^ Furthermore, lower child development scores were seen in children of mothers with depressive symptoms, low schooling, two or more children under seven years of age living in the household, and children born prematurely and with intrauterine growth restriction.^11^ However, there is scarce literature analyzing associations between child development and sleep in early childhood especially in children born during the pandemic. Understanding how sleep duration affects child development can inform better clinical or population interventions and sleep recommendations. Additionally, the World Health Organization (WHO) identifies a research gap in the effects of sleep duration on motor development, growth, and injury.^12^ Taking all this into account, this article aims to assess whether sleep duration during the period of social distancing resulting from the COVID-19 pandemic can be a determinant of poorer early childhood development.

## Materials and methods

### Study site and data

This study uses data from the Iracema-Covid, a cohort study carried out with mothers and their babies born in Fortaleza during the COVID-19 pandemic, specifically in July and August 2020.^13^ The study was approved by an Ethics Committee (73516417.4.0000.5049), and formal consent was provided by all participants. Four rounds of data collection have been conducted (6, 12, 18, and 24 months after birth). In this study, the authors use data from the third and fourth rounds (18 and 24 months after birth). Women who delivered in July or August 2020 in public hospitals in Fortaleza, aged over 18, and with complete address information were eligible for the study (*n* = 3567). A representative sample of 352 women was selected to detect a 45.7 % prevalence of maternal common mental disorders (CMDs) with a 5 % margin of error and 95 % confidence interval. An additional 371 women were randomly selected to account for potential attrition.^13^ Briefly, in the third and fourth rounds, 331 mother-child dyads (94.6 % response rate) were interviewed. Of those, 12 children presented neurological disorders that compromise child development, and 41 did not answer at least one question regarding child sleep patterns. Therefore, the authors used 278 mother-child dyads for the present analysis.

The main outcome of interest in this study is ECD, measured through the Caregiver Reported Early Development Instrument (CREDI). CREDI is a caregiver-reported, cross-culturally comparable, population-level measure of ECD for children under three years. It provides an accurate and user-friendly assessment of four domains of ECD: motor, cognitive, language, and social-emotional. CREDI has been translated and validated in Brazil and is freely available.^10^ The authors used the long version of CREDI which has been designed for large-scale research and has 117 questions with yes/no answers. Those answers are used to calculate scores for each ECD domain, as well as an overall score. In this study, the authors used CREDI scores from the fourth round of Iracema-Covid (children aged 24 months). Scores were calculated using an algorithm created by the developers of CREDI (https://credi.shinyapps.io/Scoring_App/) and are expressed as age-adjusted z-scores considering a normal distribution. The z-scores were also categorized as 〈 −2SD; −2 ≤ SD ≤ −1; −0.99 ≤ SD ≤ 0.99; 1 ≤ SD ≤ 2; and 〉 2SD for descriptive purposes.

The main effect of interest was the child's sleep duration. It was measured in the third and fourth rounds of Iracema-Covid with the Brief Infant Sleep Questionnaire (BISQ). BISQ is answered by the parents, takes 5 to 10 min, and covers the following questions with a recall period of one week: 1) nocturnal sleep duration (between 7pm and 7am); 2) daytime sleep duration (between 7am and 7pm); 3) number of night awakenings; 4) duration of wakefulness during the night hours (10pm to 6am); 5) nocturnal sleep-onset time (the time at which the child falls asleep); 6) settling time (latency to falling asleep at night); 7) method of falling asleep; 8) location of sleep; 9) preferred body position; 10) age of the child; 11) gender of the child; 12) birth order; and 13) role of the responder. The authors also considered the total daily sleep duration (hours) measured as the sum of nocturnal sleep duration and daytime sleep duration. BISQ has been translated and validated for use in Brazil.^14^ The following variables recorded missing values: nocturnal sleep duration (6 cases); daytime sleep duration (1 case); time awake at night (1 case); and number of night awakenings (6 cases).

Additional variables from Iracema-Covid were selected based on their expected relevance to ECD, including child sex, race, income, premature birth, maternal partnership status, daycare attendance, childcare assistance, maternal education, and age. Other factors considered were whether the mother received cash assistance, had private health insurance, and had other children under 6 years old.

### Statistical analysis

Continuous numerical variables were described with means and standard deviations, while categorical variables were summarized using frequencies. Data normality was assessed with the Shapiro-Wilk test. The inferential analysis employed the Spearman test to explore correlations between CREDI domain z-scores and child sleep duration variables.

Crude and adjusted linear regression models were run considering the scores of each CREDI domain as the outcome. Covariates included BISQ variables (nocturnal sleep duration; daytime sleep duration; number of night awakenings; and duration of wakefulness during the night hours), and other controls from Iracema-Covid, as described above. The authors used a backward stepwise regression so that variables that did not have significance between 0.05 and 0.20 were gradually removed. Final significance was considered for p-values < 0.05. Multicollinearity between variables was tested using the Variance Inflation Factor (VIF) with a cut-off point of 10. All statistical analyses were carried out in Stata 11.

Ethical approval and participant consent were obtained as recommended (National Research Ethics Committee in Brazil - Number 73516417.4.0000.5049) and the data are available upon request.

## Results

Mothers were on average 26.24 years old (SD: 0.95), had 10.63 years of education (SD: 2.61), were mostly black/brown (70.50 %), and were living with a partner (75.90 %). Among CREDI domains, language had the lowest score (−0.58; SD: 1.13). Children, on average, slept 9.39 h (SD: 1.46) during the night, 2.90 h (SD: 1.40) during the day, stayed 0.49 h (SD: 1.35) awake at night, slept a daily total of 12.24 h (SD: 0.95), woke up an average of 0.87 times (SD: 1.32) during the night, and most slept >11 h a day (84.53 %). Means and standard deviations (SD) of CREDI domain scores among survey participants at 24 months were: −0.35 (SD 1.20) for cognition; −0.58 (SD 1.13) for language; −0.47 (SD 0.95) for motor; −0.35 (SD 1.17) for social-emotional; and −0.50 (SD 1.23) for the overall domain (Table 1). It should be noted that the evolution of the participants' development from 18 to 24 months seems to be within an expected pattern, as can be seen from the distribution of standard deviations of the CREDI scores in the two waves (Supplementary Table1).

**Table 1:** Description of the studied sample, Iracema-COVID Cohort Study. Fortaleza, Brazil, 2021–2022.

Variables	Mean	SD	n	%
Child Development Domains (CREDI)				
Cognition (z-scores measured at 24 months)	−0.35	1.20		
> 2SD (> 2.4)			2	0.72
1 ≤ SD ≤ 2 (1.21 – 2.4)			14	5.04
−0.99 ≤ SD ≤ 0.99 (± 1.2)			209	75.18
−2 ≤ SD ≤ −1 (−1.21 – −2.4)			33	11.87
< −2SD (<−2.4)			20	7.19
Language (z-scores measured at 24 months)	−0.58	1.13		
> 2SD (> 2.2)			0	0.00
1 ≤ SD ≤ 2 (1.14 – 2.2)			13	4.68
−0.99 ≤ SD ≤ 0.99 (± 1.14)			179	64.39
−2 ≤ SD ≤ −1 (−1.14 – −2.2)			65	23.38
< −2SD (< −2.2)			21	7.55
Motor (z-scores measured at 24 months)	−0.47	0.95		
> 2SD (> 1.9)			1	0.36
1 ≤ SD ≤ 2 (0.96 – 1.9)			15	5.40
−0.99 ≤ SD ≤0.99 (± 0.96)			187	67.27
−2 ≤ SD ≤ −1 (−0.96 – −1.9)			51	18.35
< −2SD (< −1.9)			24	8.63
Social-emotional (z-scores measured at 24 months)	−0.35	1.17		
> 2SD (> 2.3)			2	0.72
1 ≤ SD ≤ 2 (1.18 – 2.3)			19	6.83
−0.99 ≤ SD ≤ 0.99 (± 1.18)			205	73.74
−2 ≤ SD ≤ −1 (−1.18 – −2.3)			35	12.59
< −2SD (< −2.3)			17	6.12
Overall (z-scores measured at 24 months)	−0.50	1.23		
> 2SD (> 2.4)			0	0.00
1 ≤ SD ≤ 2 (1.24 – 2.4)			14	5.04
−0.99 ≤ SD ≤ 0.99 (± 1.24)			199	71.58
−2 ≤ SD ≤ −1 (−1.24 – −2.4)			45	16.19
< −2SD (< −2.4)			20	7.19
Child´s Sleep Duration (measured at 18 months)				
Night sleep duration (hours)	9.39	1.46		
Daytime sleep duration (hours)	2.90	1.40		
Time awake at night (hours)	0.49	1.35		
Number of night awakenings	0.87	1.32		
Total daily sleep duration (hours)	12.23	1.92		
Child's Sleep Duration (measured at 24 months)				
Night sleep duration (hours)	9.14	1.63		
Daytime sleep duration (hours)	2.75	1.40		
Time awake at night (hours)	0.53	1.18		
Number of night awakenings	0.42	0.90		
Total daily sleep duration (hours)	11.78	2.04		
Sex of the child				
Male			142	51.08
Female			136	48.92
Premature birth			27	9.44
Child attends daycare			58	20.94
Maternal age – 24 months	26.24	0.95		
Maternal education (years) – 24 months	10.63	2.61		
Mother's skin color				
White			46	16.55
Yellow			33	11.87
Black/brown			196	70.50
Other			3	1.08
Mother lives with partner – 18 months			211	75.90
Income – 18 months (Monthly minimum wage – MMW)				
< 1 MMW			76	27.34
1 - 2 MMW			143	51.44
≥ 3 MMW			59	21.22
Mother with other sons <6 years old – 24 months			55	19.78
Mother gets help with childcare – 18 months			83	29.86
Cash transfer assistance– 24 months			175	62.95
Private health insurance			116	41.73

The authors found significant correlations between nocturnal sleep duration and all CREDI domains: cognition (0.16); language (0.14); motor (0.15); social-emotional (0.14); and overall (0.15). Similarly, total daily sleep time was correlated with the language (0.16), social-emotional (0.12), and overall (0.15) CREDI domains. Negative and significant correlations were found between time awake at night and all CREDI domains: cognition (−0.23); language (−0.15); motor (−0.21); social-emotional (−0.15); and overall (−0.17) (Table 2).

**Table 2:** Correlation between sleep duration (at 18 months) and CREDI domain z-scores (at 24 months), Iracema-COVID cohort study.

Variables	Cognition	Language	Motor	Social-emotional	Overall
Night sleep duration (hours)	0.1679*	0.1419*	0.1591*	0.1434*	0.1507*
Daytime sleep duration (hours)	0.0098	0.0960	0.0115	0.0566	0.0833
Time awake at night (hours)	−0.2317*	−0.1574*	−0.2120*	−0.1545*	−0.1705*
Number of night awakenings	−0.0738	−0.0368	−0.0777	−0.0867	−0.0505
Total daily sleep duration (hours)	0.1154	0.1667*	0.1091	0.1261*	0.1580*

Spearman correlation coefficient. *p < 0.05.

Fortaleza, Brazil, 2021–2022.

Adjusted regression models showed that the increase in night sleep duration was associated with better scores in all CREDI domains (p-values from 0.001 to 0.026). Therefore, each additional hour of nighttime sleep would increase the mean cognition score by 0.14 points; 0.10 points higher in the average of the language and motor (all *p* < 0.05) domains; 0.16 points higher on average for the socio-emotional domains; and 0.14 points higher in the average of the overall domain (all *p* < 0.05). Except for the language domain, the longest time awake at night indicated worse scores for the CREDI domains (p-values from 0.016 to 0.041). In the cognition domain, each hour awaked at night determined a decrease of 0.12 points in the mean score; for the motor domain, the mean decrease would be 0.09 points; and 0.11 for the socio-emotional and overall (all *p* < 0.05) domains.

Regarding other controls, each additional year of maternal education was associated with an increase of 0.05 (*p* = 0.044) in the cognition domain. Also, in the same domain, having another child under the age of 6 at home was associated with an increase of 0.45 points (95 %CI: 0.01; 0.81, *p* = 0.011). In the Language domain, each year of maternal age implied an increase of 0.14 points (*p* = 0.045), while female children had a higher score (*p* = 0.030). The score of the motor domain increased by 0.23 (*p* = 0.037) and 0.28 (*p* = 0.044) points if the child was female and attended daycare, respectively. In addition, having another child under the age of 6 at home determined an increase of 0.37 points (*p* = 0.044) in the Socio-emotional domain. Finally, for the Overall domain, being a female child and having a household income of 3 or more MMW were associated with an increase of 0.34 (*p* = 0.020) and 0.41 (*p* = 0.046) points on the domain, respectively (Table 3).

**Table 3:** Models of crude and adjusted linear regression analysis of child development domains, according to sleeping patterns and maternal and child characteristics.

	Crude coefficients of CREDI domains														
Variables	Cognitive			Language			Motor			Socio-emotional			Overall		
	Coef.	95 %CI	p	Coef.	95 %CI	p	Coef.	95 %CI	p	Coef.	95 % CI	p	Coef.	95 %CI	p
Night sleep duration (hours) – 18 months	**0.18**	**(0.09,0.28)**	**0.001**	**0.12**	**(0.03,0.20)**	**0.009**	**0.13**	**(0.06,0.21)**	**0.001**	**0.18**	**(0.09,0.27)**	**0.001**	**0.16**	**(0.06,0.26)**	**0.001**
Daytime sleep duration (hours) – 18 months	0.01	(−0.08,0.11)	0.781	0.06	(−0.03,0.15)	0.208	0.01	(−0.07,0.08)	0.837	0.03	(−0.06,0.13)	0.438	0.05	(−0.05,0.15)	0.333
Time awake at night (hours) – 18 months	**−0.17**	**(−0.27,−0.07)**	**0.001**	**−0.10**	**(−0.19,−0.01)**	**0.044**	**−0.13**	**(−0.21,−0.05)**	**0.001**	**−0.14**	**(−0.25,−0.04)**	**0.004**	**−0.14**	**(−0.25,−0.04)**	**0.007**
Number of night awakenings – 18 months	−0.08	(−0.19,0.02)	0.114	−0.02	(−0.12,0.07)	0.671	−0.06	(−0.14,0.01)	0.127	−0.09	(−0.20,0.01)	0.060	−0.05	(−0.16,0.05)	0.335
Sex of the child (ref: male)															
Female	**0.39**	**(0.11,0.67)**	**0.006**	**0.40**	**(0.13,0.66)**	**0.003**	**0.30**	**(0.08,0.52)**	**0.007**	**0.35**	**(0.07,0.62)**	**0.012**	**0.46**	**(0.17,0.75)**	**0.002**
Premature birth	−0.37	(−0.85,0.10)	0.125	−0.15	(−0.60,0.29)	0.508	−0.34	(−0.72,0.03)	0.076	−0.35	(−0.08,0.11)	0.135	−0.28	(−0.77,0.20)	0.254
Child attends daycare	0.08	(−0.26,0.43)	0.639	−0.11	(−0.44,0.20)	0.474	0.04	(−0.23,0.31)	0.764	−0.01	(−0.34,0.33)	0.972	−0.08	(−0.44,0.27)	0.648
Mother's age – 24 months	0.13	(−0.01,0.28)	0.067	**0.19**	**(0.05,0.33)**	**0.006**	0.04	(−0.07,0.16)	0.482	**0.17**	**(0.03,0.32)**	**0.016**	**0.16**	**(0.01,0.32)**	**0.029**
Maternal education (years) – 24 months	**0.05**	**(0.01,0.11)**	**0.033**	0.02	(−0.02,0.08)	0.263	0.02	(−0.01,0.06)	0.267	**0.05**	**(0.01,0.11)**	**0.033**	0.04	(−0.01,0.09)	0.151
Mother's skin color (ref: white)															
Black/brown	0.13	(−0.41,0.67)	0.634	0.15	(−0.35,0.66)	0.543	0.20	(−0.22,0.63)	0.347	0.10	(−0.42,0.62)	0.696	0.14	(−0.41,0.70)	0.608
Yellow	0.04	(−0.34,0.43)	0.810	0.02	(−0.33,0.39)	0.890	0.01	(−0.28,0.32)	0.903	0.06	(−0.31,0.44)	0.726	0.01	(−0.38,0.41)	0.960
Other	0.75	(−0.66,2.17)	0.296	0.73	(−0.59,2.06)	0.279	0.45	(−0.61,1.57)	0.429	0.63	(−0.75,2.02)	0.369	0.82	(−0.63,2.27)	0.267
Mother lives with partner – 18 months	0.12	(−0.11,0.65)	0.425	0.12	(−0.18,0.43)	0.421	0.24	(−0.01,0.50)	0.066	**0.35**	**(0.03,0.68)**	**0.029**	0.24	(−0.09,0.59)	0.151
Income – 18 months															
< 1 MMW	1			1			1			1			1		
1 - 2 MMW	0.18	(−0.15,0.42)	0.279	0.09	(−0.22,0.40)	0.572	0.08	(−0.17,0.35)	0.511	0.17	(−0.15,0.50)	0.286	0.14	(−0.20,0.48)	0.424
≥ 3 MMW	0.33	(−0.07,0.74)	0.107	0.23	(−0.15,0.61)	0.240	0.21	(−0.10,0.54)	0.188	0.39	(−0.01,0.79)	0.053	0.31	(−0.10,0.73)	0.141
Mother has other children <6 years– 24 months	**0.41**	**(0.06,0.77)**	**0.021**	0.30	(−0.03,0.63)	0.075	**0.31**	**(−0.23,0.03)**	**0.029**	0.34	(−0.01,0.68)	0.054	0.32	(−0.04,0.68)	0.083
Mother gets help with childcare – 24 months	−0.01	(−0.04,0.02)	0.662	−0.02	(−0.05,0.01)	0.143	−0.01	(−0.04,0.01)	0.246	−0.01	(−0.03,0.03)	0.931	−0.01	(−0.05,0.01)	0.292
Cash transfer assistance– 24 months	−0.23	(−0.52,0.06)	0.121	−0.13	(−0.41,0.14)	0.336	−0.17	(−0.40,0.05)	0.136	−0.26	(−0.55,0.02)	0.070	−0.21	(−0.52,0.08)	0.155
Private health insurance	0.17	(−0.10,0.46)	0.222	0.03	(−0.23,0.30)	0.780	0.09	(−0.13,0.32)	0.398	0.09	(−0.18,0.37)	0.517	0.08	(−0.20,0.38)	0.557
Variables	Adjusted coefficients of CREDI domains														
Prob > *F*	<0.0001			<0.0007			<0.0001			<0.0001			<0.0002		
Adjusted r2	0.11			0.07			0.08			0.11			0.08		
Beta coefficient	−3.00			−6.06			−2.00			−6.02			−5.54		
Night sleep duration (hours) – 18 months	**0.14**	**(0.04,0.24)**	**0.004**	**0.10**	**(0.01,0.19)**	**0.026**	**0.10**	**(0.03,0.18)**	**0.005**	**0.16**	**(0.06,0.25)**	**0.001**	**0.14**	**(0.04,0.24)**	**0.005**
Daytime sleep duration (hours) – 18 months	**–**			0.07	(−0.01,0.17)	0.114	**–**			0.07	(−0.02,0.17)	0.131	0.08	(−0.02,0.18)	0.113
Time awake at night (hours) – 18 months	**−0.12**	**(−0.23,0.02)**	**0.016**	−0.07	(−0.17,0.02)	0.138	**−0.09**	**(−0.17,−0.01)**	**0.021**	**−0.11**	**(−0.21,−0.01)**	**0.030**	**−0.11**	**(−0.21,−0.01)**	**0.041**
Number of night awakenings – 18 months	**–**			–			**–**			–			–		
Sex of the child (ref: male)															
Female	**0.31**	**(0.03,0.58)**	**0.029**	**0.29**	**(0.02,0.55)**	**0.030**	**0.23**	**(0.01,0.45)**	**0.037**	0.23	(−0.03,0.25)	0.086	**0.34**	**(0.05,0.62)**	**0.020**
Mother's age – 24 months	**–**			**0.14**	**(0.01,0.27)**	**0.045**	**–**			0.10	(−0.03,0.51)	0.135	0.10	(−0.04,0.25)	0.172
Maternal education (years) – 24 months	**0.05**	**(0.01,0.11)**	**0.044**	**–**			**–**			0.04	(−0.01,0.10)	0.110	–		
Income – 18 months															
< 1 MMW	1			1			1			1			1		
1 - 2 MMW	0.21	(−0.11,0.54)	0.193	0.14	(−0.15,0.45)	0.344	0.15	(−0.10,0.41)	0.236	0.22	(−0.09,0.55)	0.163	0.22	(−0.10,0.56)	0.183
≥ 3 MMW	0.26	(−0.16,0.69)	0.223	0.30	(−0.07,0.68)	0.115	0.29	(−0.01,0.61)	0.063	0.34	(−0.07,0.76)	0.104	**0.41**	**(0.01,0.82)**	**0.046**
Mother has other children <6 years– 24 months	**0.45**	**(0.01,0.81)**	**0.011**	0.26	(−0.06,0.58)	0.118	**0.28**	**(0.01,0.55)**	**0.044**	**0.37**	**(0.03,0.72)**	**0.031**	0.27	(−0.07,0.62)	0.128

## Discussion

The research findings show that sleep-related variables significantly impact early childhood development (ECD) as measured by CREDI domains, in children born during the COVID-19 pandemic. Longer nocturnal sleep positively affects overall development and CREDI scores, while prolonged wakefulness worsens cognitive, motor, and social-emotional development. In the cognition domain, better maternal education, female gender, and the presence of younger siblings enhance development. Maternal age and female gender positively influence language development, while female gender and younger siblings benefit motor development. Social-emotional development is improved by younger siblings, female gender, and income ≥ 3 MMW. These results highlight the connection between sleep and child development.

Sleep duration plays a fundamental role in the child's cognitive development, contributing positively to memory consolidation, learning process, and brain plasticity, which are bases for the development of cognition,^15^ but the mechanisms of action remain poorly defined. The authors know that REM sleep (Rapid Eye Movement) allows the organism to act by selectively restricting the growth of dendritic spines and strengthening the development of new synapses.^16^ It is assumed that there are sleep-dependent, age-related changes in the child's neurocognitive development process and that two phases could be delineated in this process. The development involves an initial period of high plasticity with synapse formation and increased myelination, followed by a phase of reduced plasticity, selective synaptic pruning, white matter growth, and stable connectivity. The first stage occurs before 2–3 years, promoting neural reorganization and learning, while the second stage, after 2–3 years, supports neural repair and maintenance.^17^ The present study confirms that longer nighttime sleep and shorter wake periods are key determinants of better cognitive development in children, consistent with existing literature. These findings highlight the importance of adequate sleep for preventing cognitive impairments.

Apparently, sleep contributes by allowing the process of memory consolidation to occur. Thus, the memories initially acquired during wakefulness are more unstable and susceptible to interference, they are consolidated during sleep and made available for a third stage of access and recall. A hypothesis that may more consistently explain the need for sleep for the language learning process concerns the fact that during the waking period, learning memories, which are more labile, are encoded by the hippocampus, but during sleep, these memories would be strengthened and transferred to the neocortical networks, favoring its fixation.^18^ Therefore, children with a longer and more regular sleep cycle would have more possibilities of retaining language learning memories.

It is already known that the motor learning process is related to sleep, favored with a more consistent process of enhancement and consolidation of motor learning.^19^ Therefore, the scientific literature already shows a relationship between adequate sleep and better performance in ambulatory locomotor tasks^20^ and in the resolution of locomotor problems.^21^ Although the literature is not definitive about it, the learning consolidation process allowed by sleep also applies to the motor learning process, facilitating the retrieval of information for the development of motor skills.^22^ Adequate nighttime sleep should be considered crucial for promoting proper motor development in children and can serve as an intervention to support optimal motor growth.

How sleep contributes to the development of social and emotional skills is still unclear. It is known that interactions between children and adults act to shape brain architecture.^23^ It may be that this molding process is facilitated by the properties of high plasticity, the establishment of synapses, increased myelination, selective synaptic pruning, increased white matter, and the stabilization of connectivity that adequate sleep provides.^17^ Therefore, it is extremely important to consider sleep patterns as crucial for monitoring children's social and emotional development. Results and existing literature suggest that sleep can also be a key factor in clinical interventions to support development.

Maternal education, female gender, and presence of other children younger than 6 years showed a relationship with better cognition scores. This might be related to a richer vocabulary of mothers with better education^24^ and interaction with older children at the home^25^ environment, which can be triggers for children's cognitive development. The present results suggest that female children had better cognitive development. This could be due to structural variations of the brain according to gender;^26^ however, socioeconomic variables may also influence this result.^27^

In addition to what has already been discussed in this article, a few words should be highlighted about the pandemic period and its influence on the duration and quality of sleep in the general population. Previously published literature shows that some factors, such as lockdown and social isolation, typical of the pandemic period, may be associated with worse quality and duration of sleep.^28^ Therefore, the reading and interpretation of the results presented here must be considered in the context to which the authors were all exposed during the COVID-19 pandemic.

The present study's data may support promoting adequate nighttime sleep during pediatric follow-ups and inform health policies encouraging sleep for optimal early childhood development (ECD). These recommendations align with the Convention on the Rights of the Child, which states: '1. States Parties recognize the right of every child to a standard of adequate living for the child's physical, mental, spiritual, moral and social development. 2. The parent(s) or others responsible for the child have the primary responsibility to secure, within their abilities and financial capacities, the conditions of living necessary for the child's development.' Additionally, they reflect the UN 2030 Sustainable Development Goals, which aim to 'ensure that all girls and boys have access to quality early childhood development, care, and preprimary education so that they are ready for primary education”.

A limitation of this study is that while the BISQ measures sleep duration, it does not provide any measure of sleep quality. Nevertheless, since these results suggest that sleep duration is associated with ECD there seems to be a gain despite a possible variation in sleep quality. In addition, the positive effects of sleep duration on ECD were measured based on a cohort of children born during the COVID-19 pandemic, whose mothers were exposed to lockdown and physical distancing measures, deteriorating mental health,^29^ and reduced health care seeking behavior,^30^ among other challenges. Major strengths include the longitudinal nature of the data, and the use of CREDI to capture different domains of ECD.

The duration of nocturnal sleep influences the cognitive, motor, and social-emotional development of children during the COVID-19 pandemic. It would be advisable to include information and sensitization on the importance of sleep duration in regular primary care, pediatric consultations, and community health agent visits to facilitate adequate child development.

## Conflicts of interest

The authors declare no conflicts of interest.

## Appendix Supplementary materials

Image, application 1
