# Supplementary material for: Effect of sleep duration on child development in Fortaleza, Northeastern Brazil
Source: J Pediatr (Rio J). 2024 Oct 22;101(2):238–46. doi: 10.1016/j.jped.2024.09.002 (PMC11889661; doi:10.1016/j.jped.2024.09.002)
Supplement: Supplementary file 1 [file mmc1.docx]

**JPED-D-23-00469 – Supplementary Material**

**Supplementary Table 1** Description of the evolution of child development according to the CREDI domains at 18 and 24 months.

| **CREDI domains** |  |  | **24 months** | |  |  |
| --- | --- | --- | --- | --- | --- | --- |
| **Cognition** |  | **< -2SD – n (%)** | **-2 ≤ SD ≤ -1 – n (%)** | **-0.99 ≤ SD ≤ 0.99 – n(%)** | **1 ≤ SD ≤ 2 – n (%)** | **> 2SD – n (%)** |
| **18 months** | **<-2SD** | 11(55.0) | 6(18.18) | 6(2.87) | 1(7.14) |  |
|  | **-2 ≤ SD ≤ -1** | 7(35.0) | 13(39.39) | 30(14.35) |  |  |
|  | **-0.99 ≤ SD ≤ 0.99** | 2(10.0) | 14(42.42) | 160(76.56) | 9(64.29) | 1(50.0) |
|  | **1 ≤ SD ≤ 2** |  |  | 13(6.22) | 3(21.43) | 1(50.0) |
|  | **> 2SD** |  |  |  | 1(7.14) |  |
| **Language** |  | **< -2SD – n(%)** | **-2 ≤ SD ≤ -1– n(%)** | **-0.99 ≤ SD ≤ 0.99 – n(%)** | **1 ≤ SD ≤ 2 – n(%)** | **> 2SD – n(%)** |
| **18 months** | **< -2SD** | 8(38.10) | 12(18.46) |  |  |  |
|  | **-2 ≤ SD ≤ -1** | 9(42.86) | 30(46.15) | 21(11.73) |  |  |
|  | **-0.99 ≤ SD ≤ 0.99** | 4(19.05) | 23(35.38) | 143(79.89) | 8(61.54) |  |
|  | **1 ≤ SD ≤ 2** |  |  | 15(8.38) | 5(38.46) |  |
|  | **> 2SD** |  |  |  |  |  |
| **Motor** |  | **< -2SD – n(%)** | **-2≤SD≤-1 – n(%)** | **-0.99≤SD≤0.99 – n(%)** | **1≤SD≤2 – n(%)** | **>2SD – n(%)** |
| **18 months** | **< -2SD** | 4(16.67) | 4(7.84) | 2(1.07) |  |  |
|  | **-2 ≤ SD ≤ -1** | 15(62.50) | 15(29.41) | 17(9.09) | 1(6.67) |  |
|  | **-0.99 ≤ SD ≤ 0.99** | 5(20.83) | 23(62.75) | 142(75.94) | 9(60.0) |  |
|  | **1 ≤ SD ≤ 2** |  |  | 26(13.90) | 3(20.0) |  |
|  | **> 2SD** |  |  |  | 2(13.33) | 1(100.0) |
| **Social-emotional** |  | **< -2SD – n(%)** | **-2 ≤ SD ≤ -1– n(%)** | **-0.99 ≤ SD≤ 0.99 – n(%)** | **1 ≤ SD ≤ 2 – n (%)** | **> 2SD – n(%)** |
| **18 months** | **< -2SD** | 8(47.06) | 6(17.14) | 6(2.93) |  |  |
|  | **-2 ≤ SD ≤ -1** | 5(29.41) | 12(34.29) | 29(14.15) |  |  |
|  | **-0.99 ≤ SD ≤ 0.99** | 4(23.53) | 17(48.57) | 159(77.56) | 12(63.16) | 1(50.0) |
|  | **1 ≤ SD ≤ 2** |  |  | 11(5.37) | 5(26.32) | 1(50.0) |
|  | **> 2SD** |  |  |  | 2(10.53) |  |
| **Overall** |  | **< -2SD – n (%)** | **-2 ≤ SD ≤ -1– n (%)** | **-0.99 ≤ SD ≤ 0.99 – n (%)** | **1 ≤ SD ≤ 2 – n (%)** | **> 2SD – n (%)** |
| **18 months** | **< -2SD** | 5(25.0) | 4(8.89) | 2(1.01) |  |  |
|  | **-2 ≤ SD ≤ -1** | 12(60.0) | 15(33.33) | 12(6.03) |  |  |
|  | **-0.99 ≤ SD ≤ 0.99** | 3(15.0) | 26(57.78) | 154(77.39) | 8(57.14) |  |
|  | **1 ≤ SD ≤ 2** |  |  | 31(15.58) | 6(42.86) |  |
|  | **> 2SD** |  |  |  |  |  |
